# Risk of Serious Bacterial and Non‐Bacterial Infections in People With MASLD

**DOI:** 10.1111/liv.70059

**Published:** 2025-03-12

**Authors:** Giovanni Targher, Herbert Tilg, Luca Valenti

**Affiliations:** ^1^ Department of Medicine University of Verona Verona Italy; ^2^ Metabolic Diseases Research Unit IRCCS Sacro Cuore—Don Calabria Hospital Negrar di Valpolicella Italy; ^3^ Department of Internal Medicine I, Gastroenterology, Hepatology, Endocrinology and Metabolism Medical University Innsbruck Innsbruck Austria; ^4^ Department of Pathophysiology and Transplantation University of Milan Milan Italy; ^5^ Precision Medicine, Biological Resource Center Unit Fondazione IRCCS Ca' Granda Ospedale Maggiore Policlinico Milan Italy

**Keywords:** bacterial infections, fatty liver, infection, infectious diseases, MASLD, metabolic dysfunction‐associated steatotic liver disease, review, serious bacterial infection

## Abstract

Metabolic dysfunction‐associated steatotic liver disease (MASLD) has become the most common chronic liver disease globally. MASLD is a multisystem disease where metabolic dysfunction plays a key role in the development of MASLD and its most relevant liver‐related morbidities and extrahepatic complications, such as cardiovascular disease, chronic kidney disease and certain types of extrahepatic cancers. Among the least examined MASLD‐related extrahepatic complications, an ever‐increasing number of observational studies have reported a positive association between MASLD and the risk of serious bacterial infections (SBI) requiring hospital admission. This risk remained significant in those studies where statistical analysis was adjusted for age, sex, ethnicity, obesity, type 2 diabetes and other common comorbidities. Notably, the incidence rates of SBI were further increased with more advanced MASLD, especially in patients with MASLD‐related cirrhosis, and were also observed for some acute viral infections, including SARS‐CoV‐2 infection, leading to severe COVID‐19. In this narrative review article, we provide an overview of the literature on (a) the recent epidemiological data linking MASLD to the risk of serious bacterial and non‐bacterial infections requiring hospital admission, (b) the putative underlying mechanisms through which MASLD may increase the susceptibility to serious infections, both directly and through the immune dysfunction associated with cirrhosis and portal hypertension, and (c) the practical and clinical implications of the increased risk of serious bacterial and non‐bacterial infections in the growing global population with MASLD.

**Abbreviations**
ACLF: acute‐on‐chronic liver failure
HDL: high‐density lipoproteins
ICD: International Classification of Diseases
KCs: Kupffer cells
LDL: low‐density lipoproteins
MAFLD: metabolic dysfunction‐associated fatty liver disease
MASH: metabolic dysfunction‐associated steatohepatitis
MASLD: metabolic dysfunction‐associated steatotic liver disease
NAFLD: non‐alcoholic fatty liver disease
SBI: serious bacterial infections
T2D: type 2 diabetes

**Summary**

- Recent observational studies link MASLD to an increased risk of serious bacterial and non‐bacterial infections requiring hospital admission.
- The magnitude of this risk parallels the underlying severity of MASLD.
- Multiple mechanisms may be implicated in the increased susceptibility to serious bacterial and non‐bacterial infections in MASLD.

## 1 Introduction

Metabolic dysfunction‐associated steatotic liver disease (MASLD), formerly known as non‐alcoholic fatty liver disease (NAFLD), has emerged as a public health threat as it currently affects over 35% of the adult population worldwide [1, 2, 3]. An estimated ~50% of the global adult population is forecasted to have MASLD by 2040 [2].

In the past decade, substantial evidence indicates that MASLD is a multisystem disease that has implications for diseases beyond the liver [2, 4]. MASLD is not only associated with major adverse liver‐related outcomes but also with an increased risk of developing fatal and non‐fatal cardiovascular events, chronic kidney disease and certain extrahepatic cancers, especially non‐liver gastrointestinal cancers [5, 6, 7, 8].

Among the least examined MASLD‐related extrahepatic complications, an ever‐increasing number of observational cross‐sectional and population‐based cohort studies have also assessed the relationship between MASLD and the risk of developing serious bacterial infections (SBI), that is, those that can lead to significant health complications and that require hospital admission, such as meningitis, sepsis, pneumonia, urinary tract infections or other SBIs.

In this narrative review, we provide an overview of the literature on (a) the recent epidemiological data linking MASLD to the risk of SBI requiring hospital admission, another leading cause of morbidity and mortality, and potentially to other non‐bacterial (viral, fungal or parasitic) infections; (b) the putative underlying mechanisms through which MASLD (and factors strongly linked with MASLD, including those associated with insulin resistance and advanced liver disease) may increase the susceptibility to infections; and (c) the possible clinical implications of the increased risk of serious bacterial and non‐bacterial infections in the growing global population with MASLD.

## 2 Epidemiological Link Between MASLD and Serious Bacterial Infections

About 15 years ago, in a retrospective cross‐sectional hospital‐based study of 347 Israeli middle‐aged individuals, Nseir et al. [9] reported for the first time that ultrasound‐detected MASLD was significantly associated with a ~threefold higher risk of recurrent bacterial infections, especially urinary tract infections. This risk of SBI requiring hospital admission remained significant even after adjusting for age, sex, obesity, type 2 diabetes (T2D), metabolic syndrome, insulin resistance and other potential confounding factors. Subsequently, in two small hospital‐based case–control studies, the same group of investigators reported that imaging‐detected MASLD was also independently associated with a higher risk of community‐acquired pneumonia [10] and *Clostridioides difficile*‐associated diarrhoea [11].

In recent years, after the publication of these pioneering studies from Israel, an ever‐increasing number of observational cross‐sectional and prospective cohort studies from different countries have examined the relationship between MASLD and the risk of developing SBI, such as pneumonia, meningitis, sepsis, urinary tract infections, abdominal/gastrointestinal infections or other serious infections requiring hospital admission.

A systematic review and meta‐analysis incorporating more than 26 million adult individuals from different countries (Israel, Croatia, Sweden and the USA) in eight observational studies (six cross‐sectional hospital‐based studies and two population‐based cohort studies, published until 1 April 2024) has recently provided further support for a significant association between MASLD (diagnosed by International Classification of Diseases [ICD] codes, imaging techniques or liver biopsy) and the risk of SBI requiring hospital admission [12].

**FIGURE 1:**
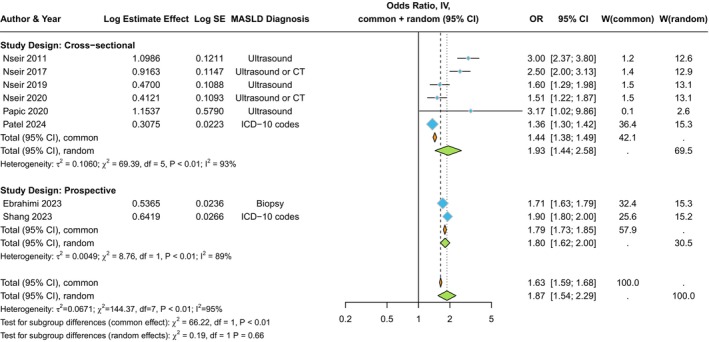
Forest plot and pooled estimates of the effect of MASLD on the risk of serious bacterial infections requiring hospital admission in the eligible studies stratified by study design (cross‐sectional vs. prospective cohort studies). Data reproduced from Mantovani et al. [12].

In this comprehensive meta‐analysis [12], MASLD was significantly associated with a ~ twofold higher risk of prevalent SBI that required in‐hospital or emergency department care (*n* = 6 studies; pooled random‐effects odds ratio 1.93, 95% CI 1.44–2.58; *I* ^*2*^ = 93%) (Figure 1). This risk remained significant in those studies where statistical analysis was adjusted for age, sex, ethnicity, obesity, T2D, Charlson comorbidity index and other clinical comorbidities. Meta‐regression analyses did not show any effect modification by age, sex, obesity or T2D on the association between MASLD and the risk of SBI. Meta‐analysis of data from the two large population‐based cohort studies from Sweden showed that MASLD (assessed by liver biopsy or ICD codes) was associated with a ~ twofold higher risk of developing incident SBI requiring hospital admission (*n* = 2 studies; pooled random‐effects hazard ratio 1.80, 95% CI 1.62–2.0; *I* ^*2*^ = 89%) (Figure 1), independent of age, sex, obesity, T2D, chronic obstructive pulmonary disease and other baseline clinical comorbidities. Notably, the incidence rates of SBI were further increased with more severe MASLD, especially with more advanced fibrosis stages, that is, bridging fibrosis and cirrhosis (pooled random‐effects hazard ratio 2.42, 95% CI 1.89–2.29) [12].

Concordant with the overall results on SBI, all subtypes of bacterial infection showed a higher risk in patients with MASLD than those without MASLD [12]. MASLD was associated with a substantially higher risk of urinary tract infections, upper or lower respiratory tract infections and gastrointestinal/abdominal infections, followed by sepsis, meningitis, peritonitis and musculoskeletal or skin infections. As reported in the two Swedish cohort studies included in the meta‐analysis [13, 14], there was a graded positive relationship between the severity of MASLD and the risk of SBI, with the highest risk observed for patients with advanced fibrosis or cirrhosis. For instance, in the nationwide cohort study by Ebrahimi et al. [13] that included 12,133 adults with biopsy‐confirmed MASLD matched 1: ≤ 5 to population comparators (*n* = 57 516) by age, sex, calendar year and county, the risk of SBI requiring hospital admission increased progressively and independently with worsening MASLD histological severity over a median follow‐up of 14.1 years. The adjusted hazard ratios were 1.64 (95% CI 1.55–1.73) for isolated steatosis, 1.84 (95% CI 1.60–2.12) for non‐fibrotic steatohepatitis, and 2.32 (95% CI 1.92–2.82) for cirrhosis. Similarly, the cohort study by Shang et al. [14] confirmed a higher risk of developing serious infections requiring hospital admission in people with more severe MASLD (diagnosed by ICD codes), independently of multiple potential confounders, over a median follow‐up of 5.3 years. The strongest risk was observed for patients with MASLD‐related cirrhosis with an adjusted hazard ratio of 3.6 (95% CI 2.7–4.6). Notably, these authors also reported that MASLD was significantly associated with a ~twofold higher risk of infection‐related mortality [14].

## 3 MASLD and Overall Risk of Bacterial and Non‐BACTERIAL Infections

The findings of our meta‐analysis complement the results of previously published meta‐analyses showing that MASLD can also increase susceptibility to non‐bacterial acute infections, such as SARS‐CoV‐2 infection leading to severe COVID‐19, especially in younger individuals [15], and in those affected by metabolic dysfunction‐associated steatohepatitis (MASH) [16], exposed to more than fourfold higher risk of severe disease, which was larger than that conferred by T2D and other metabolic comorbidities. Furthermore, these findings further support the recent results of a prospective cohort study for the CLEARED Consortium, showing that bacterial or fungal infections in hospitalised patients with cirrhosis due to MASLD or other aetiologies are associated with higher rates of in‐hospital mortality and 30‐day post‐discharge mortality (although there are significant disparities in outcomes based on country income level) [17]. A seminal study by Jalan et al. [18] also highlighted that bacterial infections play a relevant role during the clinical course of cirrhosis by precipitating or aggravating hepatic decompensation events and that any organ dysfunction other than the liver is associated with an increased risk of imminent mortality, with acute‐on‐chronic liver failure (ACLF) being the most significant expression of the disease progression.

After the publication of our comprehensive meta‐analysis on the association between MASLD and the risk of SBI requiring hospital admission [12], Zhao et al. analysed data from the Shanghai Suburban Adult Cohort and Biobank study, encompassing > 33,000 adult participants with available liver ultrasonography data who were followed for a median of 5.7 years [19]. These authors reported that ultrasound‐defined MASLD was significantly associated with an 18% higher hazard and a 5‐year absolute excess risk of ~2% for severe (bacterial, viral or parasitic) infections leading to hospitalisation or death. Among people with MASLD, those with concurrent abdominal obesity or T2D had higher incidence rates of severe infections, although the overall risk remained consistent [19]. Recently, in a nationwide retrospective observational study that included ~21 million patients admitted to hospitals in the United States between 2000 and 2019 with a primary discharge diagnosis of sepsis, Lyu et al. reported that comorbid MASLD (especially MASLD‐related cirrhosis) was associated with higher rates of in‐hospital all‐cause mortality and worse clinical outcomes in sepsis inpatients [20].

It is important to note that the findings of the observational studies mentioned above apply to SBI requiring hospital admission and, therefore, cannot be extrapolated to milder bacterial infections. Moreover, the retrospective design of these observational studies does not allow for establishing a causal association between MASLD and the risk of SBI requiring hospital admission. Although most of these studies adjusted the results for age, sex, ethnicity, obesity, T2D, and other common comorbidities, the possibility of residual confounding by unmeasured factors (e.g., food insecurity, low income, certain comorbidities, low physical activity or daily alcohol intake) cannot be entirely excluded. For example, although MASLD diagnosis requires the exclusion of significant alcohol intake, daily alcohol intake is often underreported in individuals classified as having MASLD (thus possibly inducing a misclassification bias), and metabolic risk factors and alcohol intake may synergistically interact to increase the progression of liver disease and the risk of developing bacterial infections [21]. Although all published studies have used the NAFLD nomenclature, for this review article, we have assumed that NAFLD and MASLD are synonymous, as the two fatty liver disease nomenclatures share a high level of overlap and can, therefore, be used interchangeably [22]. Conversely, because the MASLD and metabolic dysfunction‐associated fatty liver disease (MAFLD) definitions may identify different patient populations with variable disease outcomes [23, 24], it is reasonable to hypothesise that head‐to‐head MASLD/MAFLD comparative studies should be performed to ascertain the probably different risks of SBI across these two fatty liver disease nomenclatures.

## 4 Possible Mechanisms Linking MASLD to Serious Bacterial and Non‐BACTERIAL Infections

There is increasing clinical evidence that MASLD, especially when liver fibrosis evolves to an advanced stage, is associated with an increased risk of SBI requiring hospital admission. Liver diseases dependent on the stage of disease are associated with a higher rate of bacterial and fungal infections, regardless of the underlying aetiology of liver disease [25]. As reported above, food insecurity, low income, certain comorbidities, low physical activity, and daily alcohol intake could be contributing factors to the increased susceptibility for severe bacterial and non‐bacterial infections in people with advanced MASLD (especially in those who are obese or have T2D). That said, in this section, we discuss various host components more closely related to liver disease pathogenesis that may also contribute to this increased susceptibility towards infections, including important immunological players, such as macrophages, trained immunity, cytokines/inflammasomes, neutrophils, and others [25]. Gut microbiota is also involved in this susceptibility, both in early and advanced liver diseases.

### 4.1 Trained Immunity

Trained immunity is characterised by long‐term functional reprogramming of innate immune cells to combat mainly infectious diseases [26]. Some recent studies have suggested that innate immune cells residing in liver tissue in a memory effector state may be activated by consecutive exogenous stimuli (e.g., microbial or dietary components) or endogenous factors, such as metabolic signals. Clinical data on trained immunity in liver diseases or MASLD are still lacking, but it has been suggested that trained immunity might impact MASLD [27]. A recent experimental study in a zebrafish model showed that the induction of trained immunity could inhibit pyroptosis of hepatocytes to alleviate septic liver injury [28]. It is well established that human MASLD/MASH and experimental MASLD models show an increased hepatic expression of many cytokines and chemokines [29]. Both canonical and noncanonical inflammasomes play differential roles in the pathogenesis of MASLD/MASH, and various trained immunity enzymes are significantly upregulated in this condition [30]. Especially, a high‐fat, high‐cholesterol diet efficiently enhances trained immunity enzymes, suggesting that dietary components in MASLD might promote upregulation of proximal inflammasome pathways and subsequent initiation of a proinflammatory organ milieu accompanied by activation of trained immunity [30].

### 4.2 Macrophages and NK Cell Function

Liver macrophages fulfil various homeostatic functions and represent an essential line of defence against pathogenic insults [31]. The liver is frequently affected during systemic infections, and despite housing a major population of resident macrophages known as Kupffer cells (KCs), it is currently unclear whether systemic infections may permanently alter KCs and their functions. Experimental data suggest that recurrent infections may continuously reprogram KCs throughout life, potentially influencing subsequent disease susceptibility in the liver [32]. Liver macrophages and KCs are increasingly impaired as liver disease progresses towards advanced liver disease [31] due to morphological alterations in liver vascular structure [33]. In addition, circulating sulfatides are central lipids associated with fibrosis progression in MASLD, with a redistribution of high‐density lipoproteins (HDL) to low‐density lipoproteins (LDL) in more advanced liver fibrosis [34]. LDL showed a reduced content of sulfatides in liver diseases and correlated with lower activation of liver resident type II natural killer T (NKT) lymphocytes, proposing that an altered liver lipoprotein metabolism might affect liver immunity and infection susceptibility overall [34]. Therefore, it is assumed that many metabolic factors, such as certain lipids disturbed in MASLD, may impact hepatic immune cell functions.

### 4.3 Gut Microbiota

MASLD is associated with an altered gut microbiota [35]. This is especially the case in the early stages of liver disease, where dietary factors might contribute to gut dysbiosis, which worsens in the case of advanced fibrosis [36]. In this study, the authors identified proinflammatory *Proteobacteria* as potential drivers of liver disease. Gut dysbiosis has also been demonstrated to predict and be associated with mortality in solid organ transplant recipients, and mortality was primarily driven by severe infections [37]. Evidence is also increasing that bacterial DNA can be detected within the liver in patients with MASLD [38]. In an experimental mouse model of MASLD, intestinal bacteria were detected in the liver, especially in the case of steatosis, and their liver presence affected NKT cell functions, potentially influencing infection susceptibility [39]. Interestingly, *Bacteroidetes*‐derived glycosphingolipids stimulated NKT cells, promoting CCL5 signalling, thereby driving hepatic leukocyte expansion and activation [39].

Relevant modulators of gut microbiota especially reflect dietary factors, which have appeared in the past decade as one of the most important confounders of gut microbiota composition. Simple carbohydrates are, for example, able to alter gut microbiota and drive the presence of certain pathobionts, such as *Klebsiella pneumoniae* , a member of the Enterobacteriaceae family [40]. T2D is the dominant underlying disease in MASLD, and a microbiome‐based signature has been well established in T2D [41], suggesting that in many people with MASLD, gut dysbiosis might also exist at early, non‐fibrotic stages of the disease, as demonstrated by various studies [42, 43].

What remains currently unclear is how such a disturbed gut microbiota might affect overall liver immunity and increase bacterial susceptibility. However, some preclinical data have revealed important insights. The gut microbiota can regulate the differentiation of a distinct pool of RORγ^+^ regulatory T cells (Tregs) crucial for intestinal homeostasis. A recent experimental study found that microbiota‐dependent Tregs may promote tissue regeneration at extra‐gut sites, such as in injured skeletal muscle or fatty liver [44]. In a dietary MASH model, the authors of this study demonstrated that gut microbiota‐driven RORγ^+^ Tregs suppressed hepatic IL‐17 expression and liver fibrosis. Currently, it is not known whether such a phenomenon might also exist in human MASLD, but it reflects a nice example of how gut microbiota might affect liver pathology in MASLD. What affects and defines increased infection susceptibility in early liver diseases/MASLD remains unclear, and whether factors in the intestinal tract or within the liver are key driving forces also remains uncertain. It is likely that a complex interaction among microbes (gut microbiota) and intestinal and liver immunity might contribute to health and increased infection susceptibility in MASLD and other chronic liver diseases.

### 4.4 Advanced Liver Disease and Immunity

Advanced liver disease is typically characterised by a chronic inflammatory state with a massive increase in proinflammatory cytokine expression [45, 46]. Furthermore, inflammasomes are also highly upregulated in liver cirrhosis, and various other immunological factors might contribute to the immunodeficiency of this condition [47]. In cirrhosis and associated complications, patients are characterised by a profound state of immunosuppression, which is still not entirely defined as to which parts of the immune system are compromised or impaired. Besides the already mentioned players, such as trained immunity or macrophages, severe neutrophil dysfunction evolves in advanced liver diseases, which was especially demonstrated in alcoholic liver disease [48]. Interestingly, although proinflammatory cytokines are not only highly expressed in the liver tissue but also circulate at increased concentrations, isolated mononuclear cells from patients with cirrhosis exhibit a decreased synthesis of these mediators, probably also contributing to a state of immune paralysis [49]. Of crucial importance in advanced liver disease with associated portal hypertension is also an impaired intestinal barrier, which allows continuous exposure of an already immunodeficient liver to bacteria and related metabolites [50]. An impaired intestinal barrier allows the translocation of bacteria and bacteria‐derived metabolites and has long been implicated in cirrhosis‐associated immune dysfunction and infection rates [51]. The gut microbiota, which also substantially controls the intestinal barrier with its profound dysbiosis, might play a key role in cirrhosis, a condition characterised by severe intestinal dysbiosis and many pathobionts [41].

Drugs used for the treatment of certain complications in cirrhosis, such as hepatic encephalopathy, might also affect the rate of bacterial infections. Multidrug‐resistant organisms (MDR) are rapidly evolving worldwide and constitute a major problem in this patient population [52]. Rifaximin, an antibiotic that exhibits only intestinal activity, shows a clinical benefit in hepatic encephalopathy [53]. Rifaximin is widely used in these patients also in a prophylactic manner, and it has been recently demonstrated that its widespread use might have adverse consequences, causing cross‐resistance of vancomycin‐resistant *Enterococcus faecium* to daptomycin, a last‐resort antibiotic [54]. Treatment of advanced liver disease, including hepatic encephalopathy, also includes the use of prebiotic lactulose, which has the capability not only to improve patients' liver‐disease complications but is also able, by modulating the gut microbiome, to reduce the colonisation of patients with cirrhosis by multidrug‐resistant bacteria, such as vancomycin‐resistant *Enterococcus faecium* [55]. A proposed mechanism has suggested that *Bifidobacteria*, that is, a member of the physiologic gut microbiota, metabolise lactulose, producing high amounts of short‐chain fatty acids, enhancing intestinal immunity [55].

**FIGURE 2:**
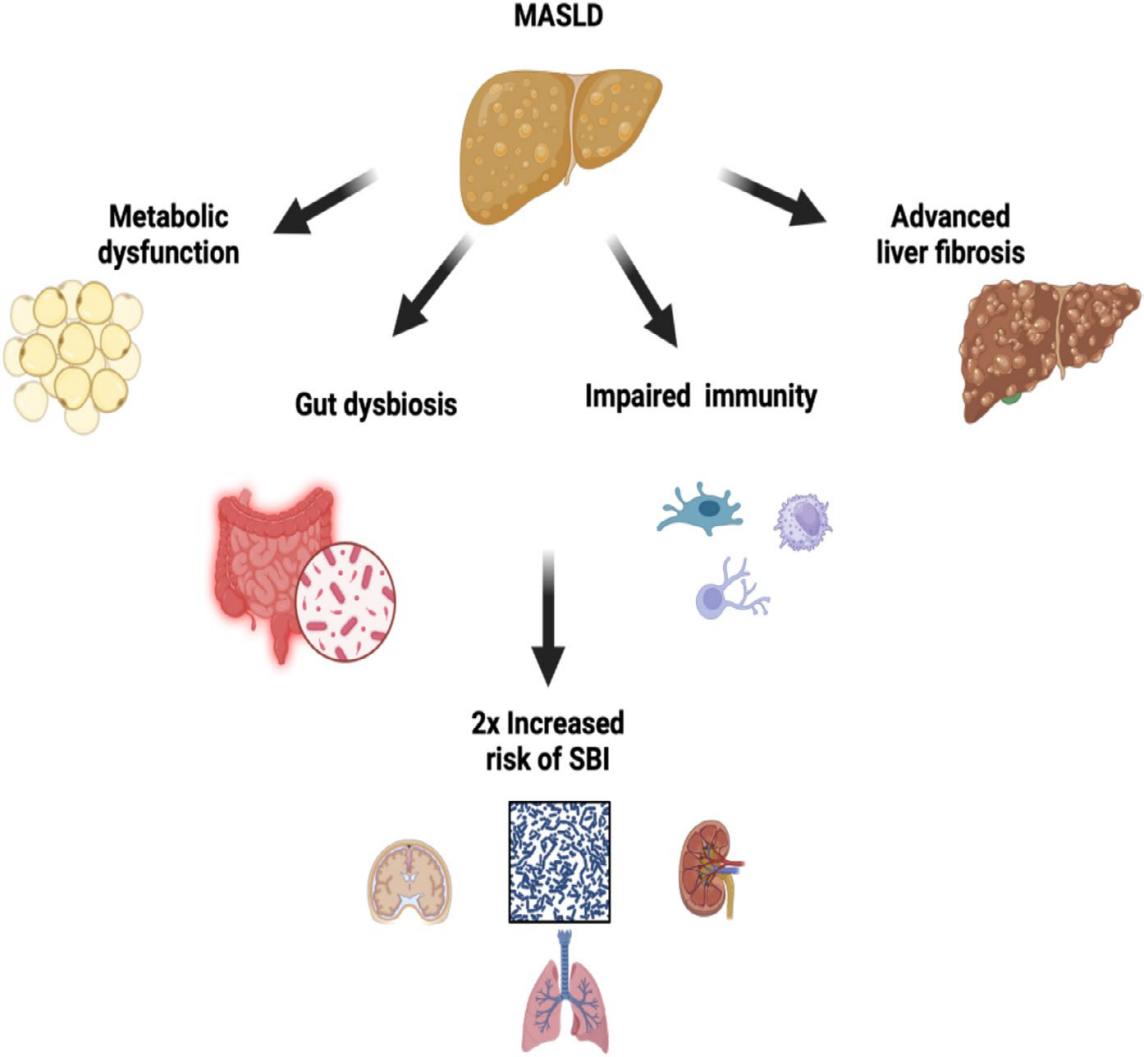
Potential underlying mechanisms linking MASLD to severe bacterial infections (SBI).

In summary, a complex network between gut and liver immunity and its microbial inhabitants can regulate immune defence in health and disease. Especially in advanced liver disease, irrespective of the underlying aetiology, many diverse players of immunity and gut microbiota may contribute to immune paralysis, driving the high susceptibility of those patients towards often fatal bacterial and non‐bacterial (fungal) infections. A more detailed discussion of potentially impaired immune mechanisms in advanced liver disease is beyond the scope of this article. A summary of the potential mechanisms linking MASLD to increased risk of SBI is schematically presented in Figure 2.

## 5 Future Directions

Further research is warranted to better define whether: (a) MASLD induces an increased susceptibility to all infections or selectively towards some specific bacterial and non‐bacterial organisms and the underlying mechanisms; (b) there is an increased risk of developing serious infection‐related complications (more clearly distinguishing this risk in cirrhotic vs. non‐cirrhotic MASLD populations); (c) the relative contribution of metabolic alterations versus the progression towards MASH, advanced fibrosis and subclinical portal hypertension, whose prevalence is most likely severely underestimated in the MASLD population; (d) there is a beneficial impact of new liver‐directed pharmacotherapies approved for fibrotic MASH (i.e., resmetirom) that can reduce hepatic fat and fibrosis on the risk of serious bacterial and non‐bacterial infections [56]; and, finally, (e) to better examine the possible impact of MASLD heterogeneity [57] on the risk of developing serious bacterial and non‐bacterial infections. Indeed, there is accumulating evidence that at least two subtypes of MASLD can exist, one associated with systemic disease and a higher risk of cardiometabolic outcomes, and the other characterised by lipid retention into hepatocytes and a more selective increase in the risk of liver disease [57, 58]. Notably, the main inherited risk variant for MASLD/MASH in the *PNPLA3* gene underlying this heterogeneity has been associated with increased hepatic secretion of proinflammatory cytokines [59], reduced susceptibility to some skin infections [60] and to severe COVID‐19 [61, 62, 63]. However, the protective impact of variant carriage in healthy or early‐stage MASLD may be offset by secondary immune dysfunction in those progressing to fibrotic MASH. Adequate consideration of sex differences, sex hormones and menopausal status in clinical investigation of the link between MASLD/MASH and the risk of SBI will also be required to fill current gaps and implement precision medicine for people with MASLD [64, 65, 66].

## 6 Current Clinical Implications

Collectively, although further studies are needed, the findings of the published observational studies summarised here strongly suggest that clinicians need to be aware that the risk of serious (bacterial and non‐bacterial) infectious diseases requiring hospital admission is high in people with MASLD and should be monitored, especially in patients with MASLD‐related cirrhosis and those who have coexisting obesity or T2D. Clinicians should also have increased clinical vigilance for SBI in this patient population and consider preventive measures, such as regular checks of vaccination status [67]. The importance of routine vaccinations for people living with advanced MASLD (especially in those with MASLD‐cirrhosis and T2D) has been elevated by the recent COVID‐19 pandemic. Routine vaccinations for pneumococcal pneumonia, influenza, hepatitis B, human papillomavirus, Herpes Zoster virus and COVID‐19 should be highly recommended to adults with advanced MASLD and coexisting T2D [68]. While taking into account the possible consequences of inappropriate empirical antibiotic therapy [69], with a ~twofold increase in the risk of SBI and infection‐related mortality [12], people with advanced MASLD should promptly receive empirical broad‐spectrum antibiotics if hospitalised for SBI, with the choice of antibiotic treatment refined when culture test results become available [67]. That said, further prospective cohort studies from different countries (especially in low‐income and lower‐middle‐income countries where the risk of SBI is higher) are needed to corroborate these results and to test whether more aggressive approaches to prevent and treat SBI might benefit people with MASLD. Mechanistic studies are also required to better elucidate the possible underlying mechanisms linking MASLD to the risk of developing serious bacterial and non‐bacterial infections requiring hospital admission. Finally, we do not believe sodium‐glucose cotransporter‐2 (SGLT2) inhibitors that may predispose to genital mycotic infections should be avoided a priori in people with MASLD and T2D because there is growing real‐world clinical evidence that the use of these glucose‐lowering drugs may significantly reduce the risk of adverse cardiovascular and renal outcomes [70], as well as likely the development of major liver‐related complications (such as hepatic decompensation events, HCC development and liver‐related deaths) in this patient population [71, 72, 73]. However, future well‐designed prospective studies in people with T2D and MASLD should also include careful monitoring of the risk of serious bacterial and non‐bacterial infections.

## 7 Conclusions

Current epidemiological evidence indicates that MASLD is significantly associated with a ~twofold increase in the risk of developing SBI requiring hospitalisation and their complications, as well as other non‐bacterial infections, including severe COVID‐19. Pending robust validation in different settings, the presence of MASLD (especially MASLD‐related cirrhosis) should probably be included among adverse factors during the patients' evaluation in clinical guidelines to prioritise more aggressive treatments. Given the high global burden of infectious diseases, the relative role of lifestyle versus metabolic versus liver‐specific factors and disease heterogeneity in determining the immune dysfunction in people with MASLD and the optimisation of clinical management should represent future research priorities.

## Author Contributions

All authors contributed equally to the manuscript. All authors edited, reviewed and approved the final version of the manuscript.

## Conflicts of Interest

The authors have no potential conflicts of interest relevant to the present manuscript to disclose. L.V. has received speaking fees from Viatris, Novo Nordisk and Boehringer Ingelheim, consulting fees from Novo Nordisk, Pfizer, Boehringer Ingelheim and Resalis and unrestricted grant support from Gilead.

## Data Availability

All supporting data of the review are available within the article.
